# Mitigating Spectral Imbalance and Detail Attenuation in RGB-Thermal Object Detection via Frequency-Guided Multimodal Fusion

**DOI:** 10.3390/s26134145

**Published:** 2026-07-01

**Authors:** Quan Du, Ming Zhao, Lu Song, Minnan Hu, Zhengqiang Wang, Wangyu Wu

**Affiliations:** 1 School of Computer Science, Yangtze University, Jingzhou 434023, China; 2024710726@yangtzeu.edu.cn (Q.D.); hitmzhao@gmail.com (M.Z.); 2 Artificial Intelligence Research Platform, Yangtze University, Jingzhou 434023, China; 3 School of Cyber Science and Engineering, Wuxi University, Wuxi 214015, China; songlu@cwxu.edu.cn (L.S.); 202312490401@nuist.edu.cn (M.H.); 4 Wuxi Key Laboratory of Artificial Intelligence and Security, Wuxi 214015, China; 5 School of Physics & Electronic Engineering, Hubei University of Arts and Science, Xiangyang 441053, China; 6 School of Computer Science, University of Liverpool, Liverpool L69 3DR, UK; wangyu.wu@liverpool.ac.uk

**Keywords:** RGB-T, multispectral detection, frequency-domain learning, cross-modal alignment, feature reconstruction

## Abstract

RGB-T object detection combines visible texture information with thermal saliency cues to improve detection under degraded illumination. Existing RGB-T fusion methods usually perform feature interaction in the spatial domain or treat spectral responses jointly, which may allow coarse background components to dominate the fusion process while weakening boundary and small-target details. In addition, the repeated upsampling and aggregation operations in the detection neck can further smooth high-frequency responses preserved during early fusion. This paper proposes F^2^Net, a frequency-guided RGB-T object detection framework built on a dual-stream YOLOv11s architecture. The method decomposes RGB and thermal features into low- and high-frequency components for separate cross-modal fusion, mitigates detail attenuation during neck decoding, and regularizes spatial correspondence between RGB and thermal representations during training. On M3FD, F^2^Net achieves 89.6% mAP@0.5 and 62.1% mAP@0.5:0.95, improving the Dual-YOLOv11s baseline by 7.7 and 6.6 percentage points, respectively, while increasing the parameter count from 13.8M to 15.4M and GFLOPs from 33.9G to 35.6G. Additional experiments on LLVIP and KAIST evaluate the method under low-light and road-scene conditions. The KAIST results show that high-IoU localization remains challenging in dense and occluded pedestrian scenes. This indicates that frequency-guided fusion mainly strengthens target response generation and moderate-IoU detection, but it does not fully solve precise boundary regression under severe occlusion and weak contour conditions.

## 1. Introduction

Object detection plays a crucial role in intelligent transportation, autonomous driving, and surveillance systems [1]. However, its performance often deteriorates under challenging illumination conditions such as darkness, fog, glare, or backlighting, where visible-light (RGB) sensors suffer from low contrast and missing texture information. In contrast, thermal infrared (TIR) imaging captures the radiation energy emitted by objects and is insensitive to lighting variations, making it highly complementary to RGB images [2]. Consequently, fusing RGB and TIR information has become a promising strategy for achieving robust all-day object detection [3].

Early RGB-T object detection methods primarily relied on straightforward feature concatenation or decision-level fusion. While these methods improved robustness to illumination changes, they often failed to capture the complex complementary relationships between the two modalities, leading to suboptimal performance. With the advancement of deep learning, multispectral detectors such as MSDS-RCNN demonstrated that adaptive weighting can effectively enhance detection accuracy [4]. Nevertheless, these models typically focus on spatial-domain fusion and ignore frequency-domain cues, which limits their ability to restore high-frequency details and fine structural information.

Recent advances in transformer-based fusion frameworks, such as the Cross-Modality Fusion Transformer (CFT), have introduced long-range dependencies to strengthen semantic interactions between modalities [5]. Despite their impressive accuracy, these models suffer from high computational cost and poor real-time performance, making them less suitable for deployment in embedded or edge devices. Lightweight architectures, such as MRD-YOLO, alleviate this issue using efficient feature aggregation, yet still struggle with modality misalignment and frequency imbalance [6].

In RGB-T detection, the benefit of dual-modal sensing depends not only on whether the two modalities are fused, but also on which components are selected during fusion and whether the fused details can survive the decoding stage. In feature-level fusion, coarse background structures usually occupy a large portion of the spectral energy, whereas target boundaries and weak local responses are often distributed in middle- and high-frequency bands. If these components are fused without distinction, background-dominated responses may suppress target-related details. Moreover, the top-down upsampling operations in the detection neck can further smooth high-frequency responses, reducing the benefit of early fusion for small or weakly salient targets. A third issue is that RGB and thermal features may interact during forward propagation but still drift in the latent feature space during training. These observations motivate a frequency-guided fusion framework that separates frequency-band interaction, compensates decoding-induced detail attenuation, and regularizes cross-modal spatial correspondence.

Experiments on M3FD [7], together with additional evaluations on LLVIP [8] and KAIST [3], demonstrate that the proposed method improves detection accuracy under complex illumination and road-scene conditions. These evaluations are used to examine cross-scene robustness rather than strict zero-shot cross-dataset generalization.

The main contributions are summarized as follows:

(1) We formulate RGB-T feature fusion from a frequency-aware sensor fusion perspective. Instead of treating fused features as homogeneous spatial responses, F^2^Net explicitly separates coarse structural responses from boundary- and detail-sensitive components during multi-scale RGB-T interaction.
(2) We design two frequency-aware operators for different stages of feature degradation. DBCM performs dual-band cross-modal modulation at backbone fusion nodes, while FISRU is designed to mitigate detail attenuation caused by neck upsampling and feature aggregation.
(3) We introduce an auxiliary spatial correspondence constraint for RGB-T feature learning and evaluate the proposed framework on M3FD, LLVIP, and KAIST. The experiments include module-level ablation and cross-scene evaluation, while the limitations in high-IoU localization and deployment efficiency are explicitly discussed.

## 2. Related Work

### 2.1. Unimodal Object Detection

Object detection has been extensively studied in computer vision and can generally be divided into RGB-based and thermal infrared (TIR)-based approaches according to the sensing modality. Early RGB-based object detection methods relied heavily on handcrafted features. A representative example is the histogram of oriented gradients (HOG) combined with a linear support vector machine (SVM) classifier, which demonstrated strong performance on early object detection benchmarks [9]. However, such traditional approaches are sensitive to occlusion, scale variation, and illumination changes, which limits their robustness in complex real-world environments.

With the rapid development of deep learning, convolutional neural network (CNN)-based detectors have gradually replaced handcrafted feature methods. Representative architectures include SSD [10], YOLO [11], and Faster R-CNN [12], which significantly improve detection accuracy and robustness by learning hierarchical feature representations. Among these methods, the YOLO series has become particularly popular due to its favorable balance between detection accuracy and real-time performance. More recently, transformer-based detection frameworks have been introduced to model long-range feature dependencies through self-attention mechanisms, further improving detection stability in complex scenes [13]. Nevertheless, RGB-based detection methods still suffer from significant performance degradation under low-light or nighttime conditions, where visual texture and color information are severely weakened.

To address the limitations of visible-light detection, TIR-based object detection has attracted increasing attention. Thermal infrared sensors capture heat radiation emitted by objects and are largely independent of ambient illumination, enabling stable perception in nighttime or low-light scenarios. Early studies explored handcrafted local descriptors for thermal object detection [14]. More recent works incorporate deep learning architectures to enhance infrared feature representation. For example, Chen et al. [2] proposed a spectrum-balanced optimization framework for thermal infrared object detection, while Chen et al. [15] introduced an attention-guided encoder–decoder convolutional neural network to improve nighttime detection performance. In addition, general-purpose detection techniques such as deformable convolution networks (DCN) [16] and hierarchical multi-scale feature representations [17] have also been adopted to enhance feature adaptability and multi-scale modeling capability in TIR imagery. Despite these advances, unimodal detectors remain constrained by either insufficient illumination robustness (RGB) or limited visual detail (TIR), motivating the development of multimodal RGB-T object detection methods.

### 2.2. Multimodal Object Detection

Multimodal object detection aims to exploit the complementary characteristics of RGB and thermal infrared modalities to improve detection robustness under diverse environmental conditions. RGB images provide rich texture and structural details, while thermal images offer stable target signatures that are less sensitive to illumination changes. Combining these two modalities can therefore significantly enhance detection performance, especially in challenging scenarios such as nighttime, adverse weather, or low-visibility environments.

A key design consideration in multispectral detection is the stage at which multimodal information is fused. Wagner et al. [18] conducted early investigations into fusion strategies and compared early and late fusion within the R-CNN framework, showing that late fusion achieved superior performance. Subsequent studies further explored intermediate feature-level fusion strategies, which enable more effective cross-modal interaction while maintaining reasonable computational efficiency. Representative examples include Halfway Fusion and Fusion RPN [19].

More recent research has focused on improving cross-modal feature representation through adaptive fusion mechanisms. Zhou et al. [20] proposed a differential modality-aware fusion (DMAF) module that dynamically selects features according to illumination conditions. AR-CNN [21] introduced a regional feature alignment (RFA) module to alleviate cross-modal misalignment caused by sensor calibration errors. Furthermore, attention-based fusion methods such as DaFF, Cross-Guided Attention, and CIAN exploit inter-modal attention mechanisms to enhance cross-modal feature correlation and improve multispectral detection accuracy [22,23,24]. Other studies have explored cross-modal aggregation strategies through multi-scale feature interaction and adaptive feature integration to better combine complementary information from RGB and thermal inputs [25]. In addition, label inconsistency and incomplete image pairings have also been addressed by approaches such as MLPD, which applies multi-label learning to handle missing modality annotations in multispectral datasets [26].

Overall, multimodal fusion methods have improved object detection performance by leveraging complementary cues from RGB and thermal modalities. However, several challenges remain, including cross-modal misalignment, spectral imbalance between modalities, and increased computational complexity introduced by sophisticated fusion mechanisms. These challenges indicate that RGB-T feature interaction still requires more explicit modeling of modality-specific information and cross-modal complementary cues.

In addition to RGB-T object detection, visible and infrared image fusion has also attracted considerable attention. Image fusion methods aim to generate a single fused image that integrates complementary information from visible and infrared modalities, thereby improving visual perception and downstream analysis tasks. For example, recent studies have explored infrared and visible image fusion using sparse representation and guided filtering in the Laplacian pyramid domain, demonstrating that frequency-aware representations can effectively enhance complementary feature integration between modalities [27].

However, fusion-based approaches typically focus on generating visually enhanced images rather than directly optimizing detection performance. In contrast, RGB-T object detection networks aim to perform feature-level fusion within end-to-end detection frameworks. Inspired by the effectiveness of frequency-domain representations in image fusion, this work introduces frequency-domain guidance into cross-modal feature interaction to enhance the complementarity between RGB and thermal features for object detection.

Although existing RGB-T detection methods have improved cross-modal feature interaction through feature-level fusion, attention mechanisms, and alignment modules, most of them still mainly operate in the spatial domain. As a result, they insufficiently distinguish low-frequency structural information from high-frequency detail cues, and the detail attenuation caused by multi-scale decoding is rarely explicitly considered. This motivates the proposed frequency-guided fusion framework, which introduces frequency-domain modulation and spectral reconstruction into RGB-T object detection.

## 3. Materials and Methods

### 3.1. Overall Network Design

**Figure 1:**
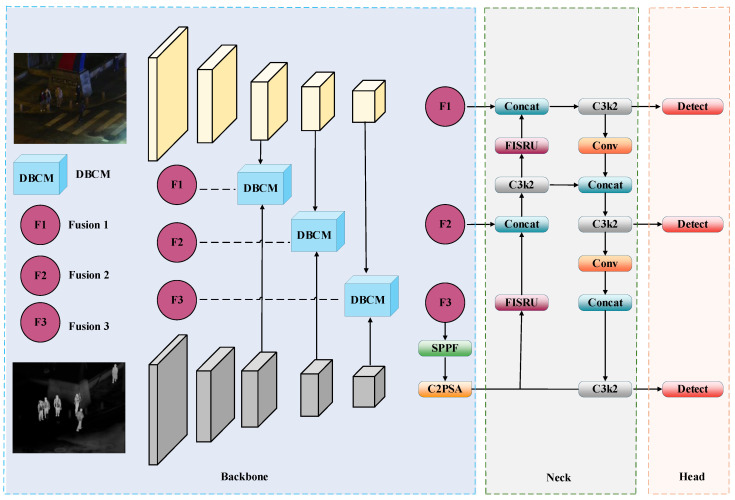
Structure of the F^2^Net model.

The overall architecture of the proposed F^2^Net is shown in Figure 1. F^2^Net is implemented on a dual-stream YOLOv11s framework, where RGB and thermal infrared images are processed by two parallel encoders. Cross-modal interaction is performed at three feature scales, denoted as F1, F2, and F3, which correspond to shallow, middle, and deep feature levels, respectively. The proposed design addresses three problems in RGB-T detection: spectral imbalance during feature fusion, high-frequency detail attenuation during neck upsampling, and insufficient optimization constraints on cross-modal feature learning.

At the backbone interaction stage, DBCM is inserted at the three scale-specific fusion nodes. Given paired RGB and thermal features, DBCM first maps them into the frequency domain and separates them into low-frequency and high-frequency components. The low-frequency path adaptively mixes structural and saliency responses from the two modalities according to local spectral reliability. The high-frequency path uses bidirectional cross-modal gating to select boundary, texture, and small-target cues while suppressing unreliable noise responses. The two frequency-band features are then reconstructed into the spatial domain and compressed into the fused feature used by the neck.

After the fused features enter the neck, repeated upsampling, concatenation, and convolutional aggregation may weaken middle- and high-frequency responses. FISRU is therefore placed after upsampling and before lateral aggregation. It uses the frequency response of bilinear interpolation as a tractable smoothing prior, applies regularized spectral compensation, and combines the reconstructed feature with the original prediction feature through learnable residual fusion. DCFL is used only during training to constrain spatial correspondence between RGB and thermal features at the same fusion scales. In this pipeline, DBCM addresses spectral imbalance during fusion, FISRU compensates decoding-induced detail attenuation, and DCFL stabilizes cross-modal feature learning.

In implementation, DBCM is inserted after the RGB and thermal backbone features at three spatial resolutions corresponding to shallow, middle, and deep stages. Each DBCM receives paired RGB and thermal feature maps with the same resolution and channel dimension and outputs a fused feature map used by the subsequent neck. FISRU is placed on the top-down reconstruction path of the neck, immediately after upsampling and before feature aggregation with the lateral feature. DCFL is applied to the projected RGB and thermal features at the same fusion scales during training only and is removed during inference. Therefore, the inference overhead comes only from DBCM and FISRU.

### 3.2. Dual-Band Complementary Modulation Module

Frequency-guided fusion in this work refers to using spectral attributes to guide both cross-modal interaction and neck reconstruction. In DBCM, RGB and thermal features are separated into low- and high-frequency bands before fusion. The low-frequency band supports structure-aware mixing, while the high-frequency band supports detail-aware cross-modal modulation. In FISRU, the frequency response of upsampling is used to estimate the components that are likely to be attenuated during neck decoding. Thus, frequency information determines both the components selected at the backbone fusion stage and the components compensated at the neck reconstruction stage.

Compared with frequency-domain fusion methods such as WaveMamba and CDDFuse, DBCM differs mainly in its use of separate modeling strategies for low-frequency and high-frequency components. Existing methods usually apply a unified mechanism after spectral decomposition, whether through attention weighting or learnable weights. Such designs do not explicitly distinguish the roles of low-frequency structural information and high-frequency detail cues in imaging and detection. For object detection, this may lead to two problems. Low-frequency background components can dominate the fusion process and suppress high-frequency responses associated with the target. The quality discrepancy between modalities within the same frequency band is also left insufficiently modeled, making a fixed fusion ratio difficult to adapt to scene dependent imaging variations. To address these issues, this study designs the dual-band complementary modulation module, DBCM, which models the low-frequency and high-frequency paths separately.

Let $F_{rgb}^{\left(l\right)},F_{tir}^{\left(l\right)}\in {\mathbb{R}}^{C\times H_{l}\times W_{l}}$ denote the feature maps produced by the RGB and thermal infrared branches at the l scale. A two-dimensional fast Fourier transform is applied to each branch to obtain its spectral representation. The resulting features are then decomposed into low-frequency and high-frequency components by a low-pass mask $M_{low}\left(\omega \right)$ and its complementary high-pass mask $M_{high}\left(\omega \right)=1-M_{low}\left(\omega \right)$. To make the frequency band partition both interpretable and smooth, the low-pass mask is defined as Equation (1).

(1) $$M_{low}(\omega )=\left\{\begin{array}{ll} 1 & ∥\omega {∥}_{2}\leq r_{c}\\ \exp\left(-\frac{{\left(∥\omega {∥}_{2}-r_{c}\right)}^{2}}{2\tau^{2}}\right), & ∥\omega {∥}_{2}>r_{c} \end{array}\right.$$

The radial frequency coordinate is normalized to [0,1] after FFT-shift. To avoid degenerate frequency masks, the cut-off radius $r_{c}$ and transition bandwidth τ are parameterized as bounded learnable scalars at each fusion scale. In our implementation, $r_{c}$ is initialized to 0.25 and constrained to [0.10,0.45], while τ is initialized to 0.05 and constrained to [0.02,0.20]. The two parameters are shared across channels within the same fusion scale and are learned independently at the shallow, middle, and deep fusion nodes. This setting allows each scale to adapt its frequency partition while keeping the mask stable and interpretable.

Here, $r_{c}$ and τ control the cut-off position and transition width of the low-pass mask. The low-frequency components mainly encode object contours and the global scene structure. Since the imaging quality of RGB and thermal infrared data varies across scenarios, direct fusion with static weights can hardly exploit their complementarity in a reliable manner. DBCM therefore introduces a quality aware mixing strategy in the low-frequency path. The local spectral energy within each spatial neighborhood is used to estimate modality quality, and is then normalized into position-wise adaptive fusion weights. The resulting low-frequency fusion is given in Equation (2).

The FFT produces complex-valued spectral features. In DBCM, the low-pass and high-pass masks are real-valued and are applied to the complex spectra by element-wise multiplication. The reliability maps and gating weights are computed from the magnitude spectra only. The generated real-valued weights are then applied to the corresponding complex coefficients, so the phase information is preserved during modulation. After inverse FFT, the real part of the reconstructed feature is used as the spatial-domain output, while the negligible imaginary part caused by numerical computation is discarded.

(2) $${\hat{F}}_{low}^{\left(l\right)}=\alpha_{rgb}^{\left(l\right)}⊙{\hat{F}}_{rgb,low}^{\left(l\right)}+\alpha_{tir}^{\left(l\right)}⊙{\hat{F}}_{tir,low}^{\left(l\right)}$$

With this design, the modality that provides more stable structural information and higher local feature reliability is assigned a larger weight at the corresponding position.

Specifically, the magnitude spectra of the low-frequency RGB and thermal features are averaged by a 3 × 3 local window to obtain modality reliability maps. A small constant ε = $1\;\times \;10^{-6}$ is used for numerical stability. The two reliability maps are normalized by softmax along the modality dimension, producing position-wise weights $\alpha_{rgb}$ and $\alpha_{tir}$, where $\alpha_{rgb}+\alpha_{tir}=1$. In this way, the modality with stronger and more coherent low-frequency responses contributes more to the fused structural representation at each frequency position.

This reduces information mismatch in low-frequency fusion and preserves the consistency of object contours and the dominant scene structure. High-frequency components contain target-related edges and texture details, but they also carry background textures, thermal noise, and pseudo responses caused by modality mismatch. A constrained form of selective enhancement is therefore required. To this end, DBCM adopts a bidirectional cross-modal gating mechanism, in which the normalized high-frequency amplitude response of each modality is passed through a gating projection and a sigmoid activation to generate bidirectional gating weights. The salient high-frequency response from one modality guides detail selection in the other modality. The normalized high-frequency magnitude response is defined as

(3) $$A_{m,h}^{\left(l\right)}=\frac{\left|\hat{F}m,h^{\left(l\right)}\right|}{\mathrm{Mean}\left(\left|\hat{F}m,h^{\left(l\right)}\right|\right)+\epsilon },\;m\in rgb,tir.$$

The bidirectional gating weights are generated from the normalized magnitude spectra:

(4) $$[G_{tir\to rgb}^{\left(l\right)}=\sigma \left(\psi_{tir\to rgb}\left(A_{tir,h}^{\left(l\right)}\right)\right),\;G_{rgb\to tir}^{\left(l\right)}=\sigma \left(\psi_{rgb\to tir}\left(A_{rgb,h}^{\left(l\right)}\right)\right)$$

The gates are real-valued and are applied to the complex-valued high-frequency spectra:

(5) $$F_{rgb,h}^{\left(l\right)}=\left(1+G_{tir\to rgb}^{\left(l\right)}\right)⊙{\hat{F}}_{rgb,h}^{\left(l\right)}$$

(6) $$F_{tir,h}^{\left(l\right)}=\left(1+G_{rgb\to tir}^{\left(l\right)}\right)⊙{\hat{F}}_{tir,h}^{\left(l\right)}$$

where ψ(·) denotes a 1 × 1 convolution, σ(·) denotes the sigmoid function, and ⊙ denotes element-wise multiplication.

After the low-frequency fusion spectrum and the enhanced high-frequency fusion spectrum are obtained, the two frequency bands are transformed back to the spatial domain by inverse Fourier transform. The real-valued low- and high-frequency features are then concatenated and compressed by a 1 × 1 convolution to obtain the fused feature. A spectral channel recalibration block, implemented with global average pooling and a two-layer fully connected mapping, further assigns channel-wise weights to detection-relevant responses. The enhanced high-frequency fusion and final output are defined in Equations (7) and (8).

(7) $${\hat{F}}_{h}^{\left(l\right)}={\tilde{F}}_{rgb,h}^{\left(l\right)}+{\tilde{F}}_{tir,h}^{\left(l\right)}$$

(8) $$F_{out}^{\left(l\right)}=s^{\left(l\right)}⊙F_{fusion}^{\left(l\right)}$$

**Figure 2:**
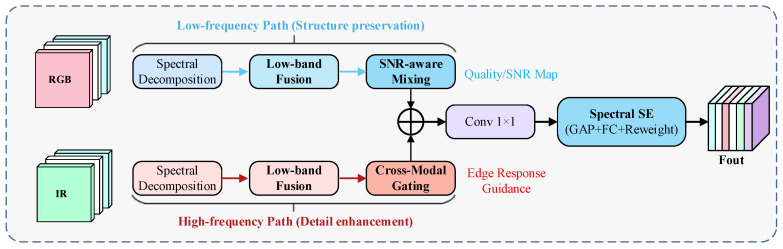
Architecture of the DBCM module. Magnitude spectra are used to estimate reliability maps and cross-modal gating weights, while the generated real-valued weights are applied to complex-valued spectra. Phase information is preserved during modulation, and the spatial-domain fused feature is reconstructed by IFFT.

The key to DBCM is therefore not merely the use of weighting, but the placement of weighting after frequency attributes have been separated. The low-frequency path focuses on structural preservation, whereas the high-frequency path emphasizes detail enhancement. These two paths are processed separately and then merged. This design reduces the masking effect of low-frequency background components on high-frequency discriminative cues in holistic spatial fusion, and provides cross-modal fused features that are better suited to object detection in the subsequent decoding stage. The overall structure of DBCM is shown in Figure 2.

### 3.3. Detail Reconstruction Module Guided by Frequency-Domain Information

DBCM performs cross-modal frequency complementary fusion during feature interaction, but whether the fusion gain can reach the detection head still depends on the decoding process in the neck. The top-down reconstruction path increases feature resolution, but it may also weaken boundary-sensitive and small-scale responses during repeated upsampling and aggregation. This attenuation is not attributed to upsampling alone, since concatenation, convolution, and feature aggregation also change the feature distribution. In this work, we focus on the dominant smoothing tendency introduced during neck reconstruction and design FISRU as a frequency-informed compensation unit. FISRU does not fuse the two modalities again. It refines the existing fused features by compensating for detail-related components that may be weakened during decoding.

Let $L^{\left(l\right)}$ denote the low-resolution feature map to be reconstructed at the$\;\left(l\right)$th scale in the neck, and let $X_{0}^{\left(l\right)}$ be the initial prediction feature generated by the corresponding branch. FISRU regards decoding as a degraded mapping with spectral smoothing, and compensates for this degradation in the frequency domain through inverse filtering. A two-dimensional fast Fourier transform is applied to $L^{\left(l\right)}$ to obtain its spectral representation ${\hat{L}}^{\left(l\right)}$. Meanwhile, the power spectrum of $X_{0}^{\left(l\right)}$ is estimated as defined in Equation (9).

(9) $$B^{\left(l\right)}={\left|F\left(X_{0}^{\left(l\right)}\right)\right|}^{2}$$

To describe the smoothing tendency of the explicit upsampling step, we use the frequency response of bilinear interpolation as a tractable prior. This approximation is not intended to represent the complete YOLO neck. Concatenation, convolution, and feature aggregation also reshape the feature spectrum and may change local responses. Therefore, FISRU does not attempt to invert the entire neck transformation. It uses the bilinear response only as a tractable frequency prior to guide the compensation of middle- and high-frequency components that are closely related to object boundaries and small-scale targets.

Let s denote the upsampling factor and ω the frequency variable. Bilinear interpolation can be approximated as the action of a separable triangular interpolation kernel in the spatial domain, whose frequency response has a low-pass property, as defined in Equation (10).

(10) $$H(\omega_{x},\omega_{y})={\left(\frac{\sin(\pi \omega_{x}/s)}{\pi \omega_{x}/s}\right)}^{2}\cdot {\left(\frac{\sin(\pi \omega_{y}/s)}{\pi_{y}/s}\right)}^{2}$$

Here,$\;\omega_{x}\;$and $\omega_{y}$ denote the normalized frequencies along the horizontal and vertical directions. In the frequency domain, bilinear interpolation is equivalent to the product of separable triangular windows. Its response approaches unity in the low-frequency range and decreases monotonically as the spatial frequency increases, with particularly strong attenuation in the middle and high-frequency bands. This property indicates that upsampling continuously compresses the discriminative components associated with small object edges and contours, which are mainly distributed in the middle and high-frequency ranges. The inverse filtering weights in FISRU are therefore designed to compensate for this attenuated region. Although the frequency response of bilinear interpolation may incur modeling errors near the Nyquist frequency, the discriminative frequency components of small objects after feature extraction are usually located in the middle and high-frequency bands rather than in the extreme high-frequency range. The approximation thus provides a reasonable estimate of attenuation for target-related frequencies. The power spectrum regularization term further limits the risk of excessive amplification of extreme high-frequency noise, allowing detail restoration and noise suppression to remain balanced. The inverse filtering weight is given in Equation (11).

(11) $$W_{freq}(\omega )=\frac{H^{*}(\omega )}{|H(\omega ){|}^{2}+\lambda P(F_{pred}^{\left(l\right)})+\varepsilon }$$

where $H\left(\omega \right)$ denotes the frequency prior derived from the upsampling response, $H^{*}\left(\omega \right)$ is its conjugate spectrum, λ is the regularization coefficient, and ε is a stability term. In this formulation, $W_{freq}\left(\omega \right)\;$is not treated as the exact inverse of the whole neck. It is used as a regularized compensation weight that enhances attenuated middle- and high-frequency responses while limiting excessive amplification of noise. After the weight is applied to ${\hat{L}}^{\left(l\right)}$, an inverse Fourier transform maps the result back to the spatial domain, producing the reconstructed feature$\;F_{sr}^{\left(l\right)}$.

To balance detail recovery and semantic fidelity, and to avoid over sharpening or local pseudo enhancement under low quality inputs or cluttered backgrounds, the final output adopts learnable residual fusion, as formulated in Equation (12).

(12) $$F_{out}^{\left(l\right)}=\alpha F_{sr}^{\left(l\right)}+(1-\alpha )F_{pred}^{\left(l\right)}$$

**Figure 3:**
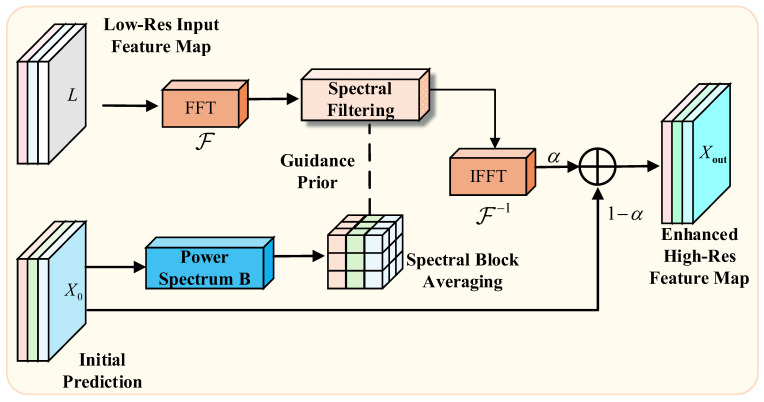
Architecture of the FISRU module.

Here, $\alpha \in \left[0,1\right]$ is a learnable fusion coefficient that adaptively controls the contribution of frequency-domain reconstruction according to the degradation level at different scales and in different scenes. This design maintains a balance between restoring local details and preserving semantic consistency. Within the overall method, DBCM produces spectrally balanced cross-modal fused features at the front-end, whereas FISRU preserves their high-frequency details during decoding. The two modules are sequentially connected but serve different purposes. The overall structure of FISRU is shown in Figure 3.

### 3.4. Dual-Constraint Fusion Loss Function

DBCM and FISRU improve cross-modal feature interaction and detail reconstruction during forward propagation. However, structural modules alone do not guarantee that RGB and thermal representations maintain stable spatial correspondence during training. For registered RGB-T image pairs, features at the same spatial location are expected to share target-related semantics, while features from different positions should remain distinguishable. To impose this constraint, we introduce a cross-modal correspondence regularization loss, denoted as DCFL. The loss contains a saliency-weighted alignment term and a spatial correspondence contrastive term. The former reduces feature drift at target-relevant locations, while the latter improves the separability of non-corresponding spatial positions. It should be noted that DCFL regularizes spatial correspondence under normal registration, but it is not designed as a geometric alignment module for severe sensor displacement, parallax, or spatial distortion.

Let the RGB and thermal infrared features at the (l)th scale be projected by a shared embedding function $\Phi \left(\cdot \right)$, yielding the latent representations $\Phi_{rgb,i}$ and $\Phi_{tir,i}$, where i indexes the spatial position. The complete form of DCFL is defined in Equation (13).

(13) $$L_{DCFL}=L_{align}+\beta L_{comp}$$

Here, $L_{align}$ denotes the modality alignment loss, $L_{comp}$ denotes the spatial correspondence contrastive loss, and β is the balancing coefficient.

To focus the constraint on salient regions that are more relevant to detection, rather than applying it uniformly across all spatial locations, the alignment loss incorporates an adaptive spatial weighting mechanism based on saliency responses. Specifically, $\omega_{i}$ is the saliency weight at the (i)th spatial location, while $\Phi_{rgb,i}$ and $\Phi_{tir,i}$ denote the RGB and thermal embeddings at the same spatial position. The formulation is given in Equation (14).

(14) $$L_{align}=\frac{1}{N}\sum_{i}\omega_{i}{\left\|\Phi_{rgb,i}-\Phi_{\mathrm{tir},i}\right\|}_{2}^{2}$$

Since complementarity in object detection is mainly reflected in local structures and high-frequency cues, an InfoNCE style contrastive loss is adopted. The RGB and thermal infrared embeddings at the same spatial location are treated as a positive pair, whereas thermal infrared features from other positions are regarded as negative samples. The loss is defined in Equation (15).

(15) $$L_{comp}=-\frac{1}{N}\sum_{i=1}^{N}\log\frac{\exp\left(\mathrm{sim}\left(\Phi_{\mathrm{rgb},i},\Phi_{\mathrm{tir},i}\right)/\tau \right)}{\sum_{j=1}^{N}\exp\left(\mathrm{sim}\left(\Phi_{\mathrm{rgb},i},\Phi_{\mathrm{tir},j}\right)/\tau \right)}$$

where $\mathrm{sim}\left(\cdot ,\cdot \right)$ denotes cosine similarity and τ is the temperature coefficient.

This objective encourages RGB and thermal embeddings at the same spatial position to be closer than embeddings from different positions. Therefore, it mainly regularizes spatial correspondence rather than explicitly maximizing modality-specific complementarity. The preservation of modality-specific cues is primarily maintained by the separate RGB and thermal branches and the frequency-band-specific design of DBCM. The two constraints play complementary roles. $L_{align}$ ensures semantic correspondence in the fused features, whereas $L_{comp}$ preserves informative modality differences. Their joint effect guides the network toward a stable balance between consistency and complementarity, forming an optimization loop that allows the complementary relations learned by DBCM and FISRU to remain effective during training.

## 4. Results

### 4.1. Experiment Settings

All experiments were conducted on a workstation running Windows 11 (Microsoft Corporation, Redmond, WA, USA) equipped with an NVIDIA GeForce RTX 4060 Ti GPU with 16 GB memory (NVIDIA Corporation, Santa Clara, CA, USA). The models were implemented using PyTorch 2.2.2. GPU acceleration was performed using CUDA 12.1 (NVIDIA Corporation, Santa Clara, CA, USA). No chemical agents or reagents were used in this study.

All input images were resized to 640 × 640. Unless otherwise specified, models were trained for 200 epochs with a batch size of 16 using stochastic gradient descent. The initial learning rate was set to 0.001, with a momentum of 0.937 and a weight decay of 0.0005. Cosine annealing was used to decay the learning rate to 0.01 of its initial value, and a linear warm-up was applied during the first three epochs.

The augmentation pipeline included Mosaic augmentation, random horizontal flipping, HSV color perturbation, and random affine transformation. All applicable models were initialized with the same COCO-pretrained weights. During inference, the confidence threshold was set to 0.001 and the IoU threshold for non-maximum suppression was set to 0.65.

For reproducibility and fair comparison, all comparative results reported in this study were reproduced under the same experimental protocol. The compared methods use the same image-pair split, 640 × 640 input resolution, training epochs, data augmentation strategy, pretrained initialization where applicable, and evaluation script. No official results from previous papers are mixed into the main comparison tables.

The YOLO-family baselines are implemented in the same code framework and trained under the same experimental protocol. YOLOv11s uses the C3k2, SPPF, and C2PSA settings adopted in our implementation. The YOLOv11 reference is used to document the architectural background, while all YOLO-family results are reproduced under the unified protocol described in this section. The same input size, training schedule, pretrained initialization, and evaluation script are used for all YOLO-family baselines.

### 4.2. Dataset

The experiments are conducted on three RGB-T benchmarks with different evaluation focuses. M3FD is used as the primary benchmark for multi-class RGB-T object detection, since it contains multiple object categories, including pedestrians, cyclists, cars, buses, and trucks, under diverse illumination and weather conditions. LLVIP and KAIST are used as pedestrian-oriented RGB-T benchmarks to further evaluate the effectiveness of the proposed fusion strategy in low-light and road-scene scenarios. Therefore, the experimental design covers both multi-class RGB-T object detection and pedestrian-focused cross-scene evaluation.

For all three datasets, the RGB and thermal images are split at the image-pair level to ensure that each RGB-T pair remains in the same subset. Each dataset is divided into training, validation, and test sets according to a 6:2:2 ratio. The same split files are used for F^2^Net and all reproduced baselines. No image pair appears in more than one subset. The validation set is used for model selection and hyperparameter tuning, while the test set is used only for final performance evaluation.

The M3FD dataset [7] contains 4200 accurately registered RGB and thermal infrared image pairs, with 34,407 annotated objects across categories such as pedestrians, cyclists, cars, buses, and trucks. In this study, M3FD is divided into 2520 training pairs, 840 validation pairs, and 840 test pairs. It covers a wide range of challenging imaging conditions, including daytime, nighttime, fog, and strong illumination interference. Its rich scene variation and clear scale diversity make it well suited for evaluating the robustness, detail preservation, and cross-modal information utilization of dual-modal detection models. It therefore serves as the core experimental platform in this study.

The LLVIP dataset [8] contains 16,836 strictly synchronized visible and thermal infrared image pairs and is mainly designed for object detection in low-illumination environments. Following the 6:2:2 splitting protocol, LLVIP is divided into 10,102 training pairs, 3367 validation pairs, and 3367 test pairs. Nighttime and dim-light scenes account for a large proportion of this dataset. LLVIP is thus used to examine how thermal infrared information contributes to modeling salient target regions when visible cues are degraded, and how the proposed fusion module improves detection under illumination deterioration.

The KAIST multispectral object detection dataset [3] contains 95,328 RGB and thermal infrared image pairs, covering representative road scenes in both daytime and nighttime conditions. According to the same 6:2:2 protocol, KAIST is divided into 57,197 training pairs, 19,066 validation pairs, and 19,065 test pairs. It includes many samples with occlusion, dense target distribution, and background interference, and is one of the most widely used benchmarks in multispectral object detection. Compared with M3FD and LLVIP, KAIST is more representative in terms of scene continuity, occlusion complexity, and road environment variation, and is used to further examine the cross-scene robustness of the proposed method in dual-modal detection tasks.

This study does not assume that all targets in the RGB-T datasets are tiny objects. The focus is placed on difficult cases where targets are relatively small, local details are weak, and foreground-background separability is reduced by low illumination, occlusion, or low saliency. M3FD provides multi-class RGB-T scenes for overall evaluation, LLVIP mainly reflects low-light pedestrian detection, and KAIST contains many dense and occluded road-scene pedestrians. These datasets therefore allow the proposed method to be examined from complementary aspects, including weak target response, low-light robustness, dense-scene detection, and occlusion tolerance.

### 4.3. Evaluation Metrics

To comprehensively evaluate the detection performance of the proposed method, several standard metrics widely used in object detection benchmarks are adopted, including precision (P), recall (R), Average Precision (AP), and mean Average Precision (mAP). These evaluation metrics follow the protocols used in the PASCAL VOC and MS COCO benchmarks [28,29].

Specifically, the detection results are categorized into true positives (TP), false positives (FP), and false negatives (FN). A true positive indicates that a predicted bounding box correctly matches a ground-truth object with an Intersection over Union (IoU) higher than a predefined threshold, while a false positive corresponds to an incorrect detection and a false negative denotes a missed object. These definitions follow commonly used evaluation strategies in object detection literature [30].

Precision measures the proportion of correctly predicted positive samples among all predicted positives, while recall measures the proportion of correctly predicted positive samples among all actual positives. They are defined as follows:

(16) $$P=\frac{TP}{TP+FP}$$

Recall represents the proportion of correctly detected positive samples among all actual positives, and is calculated as:

(17) $$R=\frac{TP}{TP+FN}$$

where TP, FP, and FN denote the numbers of true positives (correct detections), false positives (incorrect detections), and false negatives (missed detections), respectively.

Average Precision (AP) evaluates the overall detection performance by computing the area under the precision–recall curve, which is widely used in the PASCAL VOC evaluation protocol. It can be formulated as:

(18) $$AP=\int_{0}^{1}P(R)dR$$

The mean Average Precision (mAP) is obtained by averaging the AP values over all object categories:

(19) $$mAP=\frac{1}{C}\sum_{i=1}^{C}AP_{i}$$

where C denotes the total number of categories.

In this work, we report both mAP@0.5 (with an IoU threshold of 0.5) and mAP@0.5:0.95 (averaged over IoU thresholds from 0.5 to 0.95), following the evaluation protocol widely adopted in the MS COCO benchmark [30]. The former metric evaluates general detection performance, while the latter provides a more rigorous assessment of localization precision.

In addition to parameters and GFLOPs, real inference latency, FPS, and peak GPU memory consumption are reported to evaluate the practical deployment cost of the proposed method. This is necessary because FFT and IFFT operations may introduce runtime and memory overhead that cannot be fully reflected by GFLOPs.

### 4.4. Ablation Study on the Weight Parameter β

To investigate the effect of the trade-off parameter β in DCFL, a sensitivity study was conducted on the M3FD dataset using Dual-YOLOv11s as the baseline framework. Table 1 reports the loss components and detection performance under different β values, while all other training settings were kept unchanged.

When β = 0, the contrastive correspondence term is excluded, and the model is optimized mainly by the saliency-weighted alignment term. Under this setting, detection performance is limited, indicating that alignment alone is insufficient for robust cross-modal representation learning. As β increases, the contrastive correspondence term provides additional supervision for distinguishing correct cross-modal spatial correspondences from non-corresponding positions, leading to improved detection performance. The best result is obtained when β = 0.4, where mAP@0.5 and mAP@0.5:0.95 reach 0.855 and 0.680, respectively. Further increasing β leads to performance degradation, suggesting that an overly strong contrastive term may weaken the stability of fused representations for classification and localization. Therefore, β is set to 0.4 in the subsequent experiments. Since this sensitivity analysis follows the same reported experimental setting as the other results, further repeated runs with different random seeds can be used to evaluate the statistical stability of this choice.

### 4.5. Ablation Experiment

To examine the individual contributions and cooperative necessity of DBCM, FISRU, and DCFL, Dual YOLOv11s was used as the baseline, and the three modules were introduced progressively along the fusion, reconstruction, and constraint pipeline. Ablation experiments were conducted on M3FD under the same training protocol. Table 2 reports the results on M3FD.

The ablation results show that the three components contribute at different stages of the detection pipeline. DBCM brings a clear improvement over the dual-stream baseline, indicating that separating coarse structural responses from high-frequency detail cues is more effective than direct feature interaction. Its gain is larger on mAP@0.5 than on mAP@0.5:0.95, which suggests that frequency-band modulation mainly strengthens target response generation and foreground-background discrimination when used alone.

FISRU produces a single-module improvement over the Dual-YOLOv11s baseline. This result provides indirect empirical support that detail attenuation during neck reconstruction may affect detection performance. The improvement should not be interpreted as evidence that upsampling alone causes all degradation. Rather, it suggests that the whole top-down reconstruction process, including upsampling, concatenation, and convolutional aggregation, may weaken part of the boundary-sensitive responses. Under this setting, frequency-informed compensation helps preserve local details that are useful for small or weakly salient targets.

The strongest two-module setting is obtained by combining FISRU with the DCFL. This is an important observation: reconstructed high-frequency cues become more useful when RGB and thermal features are constrained to maintain spatial correspondence during training. After DBCM is further added, the complete F^2^Net obtains the best overall accuracy, showing that frequency-band fusion, detail reconstruction, and correspondence regularization are complementary rather than redundant.

To directly examine whether the gain of FISRU comes from frequency-informed reconstruction rather than ordinary spatial upsampling, an additional comparison was conducted under the same M3FD setting. DBCM and DCFL were not used in this comparison, so that the effect of the neck reconstruction operation could be isolated. The backbone, input size, training schedule, data split, and evaluation script were kept unchanged. Only the reconstruction operation in the neck was changed. Nearest-neighbor upsampling denotes an independently trained ordinary spatial reconstruction setting without frequency compensation. Bilinear upsampling replaces the original interpolation operation with bilinear interpolation. FISRU is inserted at the same reconstruction position and uses regularized frequency compensation.

As shown in Table 3, FISRU achieves the best precision, recall, mAP@0.5, and mAP@0.5:0.95 under the same training protocol. Compared with nearest-neighbor upsampling, FISRU improves mAP@0.5 by 0.9 percentage points and mAP@0.5:0.95 by 0.9 percentage points. Compared with bilinear upsampling, FISRU further improves mAP@0.5 by 0.3 percentage points and mAP@0.5:0.95 by 0.4 percentage points. This result indicates that the gain of FISRU does not come from ordinary spatial interpolation alone. By introducing regularized frequency compensation, FISRU better preserves detail-related responses during neck reconstruction and provides more effective support for boundary-sensitive and weak small-target regions, with only a small increase in parameters and GFLOPs.

The additional computational cost is moderate in terms of parameters and GFLOPs. Since F^2^Net contains FFT and IFFT operations, real inference latency, FPS, and peak GPU memory are further reported on the same RTX4060Ti platform. This evaluation provides a more direct estimate of the practical overhead introduced by DBCM and FISRU.

The real inference efficiency is measured on the same RTX4060Ti GPU used in the experiments. All models were tested with batch size 1 and 640 × 640 input resolution. Before timing, 100 warm-up iterations were performed, and the latency was averaged over 1000 inference runs. CUDA synchronization was used before and after each run to ensure accurate timing. Peak memory consumption was recorded during inference using the same testing configuration. The results are reported in Table 4.

Compared with Dual-YOLOv11s, F^2^Net increases latency from 8.7 ms to 9.3 ms and reduces FPS from 115 to 108. Peak memory consumption increases from 2486 MB to 2619 MB. The additional latency is 0.6 ms, and the memory increase is 133 MB. These results show that FFT and IFFT introduce extra runtime and memory cost, while F^2^Net still improves detection accuracy with a moderate efficiency cost on the RTX4060Ti platform.

### 4.6. Comparative Experiment

This section evaluates F^2^Net from three perspectives. First, RGB-only and IR-only detectors were used as single-modal references to examine whether paired RGB-T sensing provides additional information beyond either modality alone. Second, F^2^Net was compared with dual-stream baselines and recent RGB-T detection methods on M3FD, which is the primary multi-class benchmark in this study. Third, additional evaluations on LLVIP and KAIST were conducted to examine the method under low-light and pedestrian-oriented road-scene settings. The LLVIP and KAIST experiments are not zero-shot cross-dataset tests; each dataset is trained and evaluated under its own split. Table 5 reports the single-modal reference results on M3FD. Among the RGB-only models, RT-DETR obtains the highest accuracy, with 87.2% mAP@0.5 and 58.8% mAP@0.5:0.95. Among the IR-only models, RT-DETR also performs best, reaching 83.4% and 55.6%, respectively. F^2^Net uses paired RGB and thermal inputs and achieves 89.6% mAP@0.5 and 62.1% mAP@0.5:0.95. Compared with the strongest RGB-only reference, the gains are 2.4 and 3.3 percentage points. Compared with the strongest IR-only reference, the gains are 6.2 and 6.5 percentage points. These results show that dual-modal sensing provides useful information beyond either single modality under the evaluated M3FD protocol. However, this comparison should be interpreted as a modality reference rather than a strict architectural comparison, because F^2^Net uses two input streams and additional fusion modules.

Table 6 compares F^2^Net with dual-stream baselines and reproduced RGB-T detection methods on M3FD. Since all methods follow the same split, input resolution, training schedule, augmentation setting, pretrained initialization where applicable, and evaluation script, the comparison mainly reflects the effect of model design under a unified protocol. Compared with Dual-YOLOv11s, F^2^Net improves mAP@0.5 from 81.9% to 89.6% and mAP@0.5:0.95 from 55.5% to 62.1%. This gain indicates that the improvement is not simply caused by using paired RGB-T inputs, but is related to the proposed frequency-guided fusion, spectral reconstruction, and correspondence regularization. Compared with recent dual-modal methods, F^2^Net achieves a favorable accuracy-complexity trade-off. The margin over WaveMamba is 0.5 percentage points in mAP@0.5 and 1.3 percentage points in mAP@0.5:0.95, so small differences among top-performing methods should still be interpreted cautiously.

Table 7 reports additional evaluations on LLVIP and KAIST. These experiments are used to examine dataset-specific robustness rather than zero-shot cross-dataset generalization. LLVIP mainly reflects low-light pedestrian detection, where visible cues are often degraded and thermal saliency becomes important. On this dataset, F^2^Net achieves 95.2%mAP@0.5 and 62.3%mAP@0.5:0.95. The improvement over WaveMamba is limited, indicating that the benefit of frequency-aware fusion becomes smaller when pedestrians already have strong thermal saliency. KAIST contains dense and occluded road-scene pedestrians. On this dataset, F^2^Net achieves 76.7%mAP@0.5 and 33.8%mAP@0.5:0.95. The gap between mAP@0.5 and mAP@0.5:0.95 shows that F^2^Net improves target response generation, but precise localization remains difficult under severe occlusion and dense distribution. This is consistent with the proposed design. Frequency separation mainly strengthens saliency and detail-related cues, while high-IoU localization still depends on complete contours and accurate boundary regression.

The comparisons in Table 5, Table 6 and Table 7 should be interpreted at three levels: single-modal reference, dual-stream baseline comparison, and comparison with recent RGB-T detection methods. The single-modal results in Table 5 show that paired RGB-T inputs provide useful information beyond either RGB or thermal input alone. On M3FD, F^2^Net exceeds the strongest RGB-only reference, RT-DETR, by 2.4 percentage points in mAP@0.5 and 3.3 percentage points in mAP@0.5:0.95, and exceeds the strongest IR-only reference, RT-DETR, by 6.2 and 6.5 percentage points. Within the YOLO-family baselines, F^2^Net improves over RGB-only YOLOv11s by 8.0 and 6.9 percentage points, and over IR-only YOLOv11s by 9.8 and 8.1 percentage points. Compared with Dual-YOLOv11s, F^2^Net improves mAP@0.5 and mAP@0.5:0.95 by 7.7 and 6.6 percentage points, indicating that the gain is related to both complementary RGB-T sensing and the proposed frequency-guided design.

### 4.7. Detection Result Visualization Analysis

**Figure 4:**
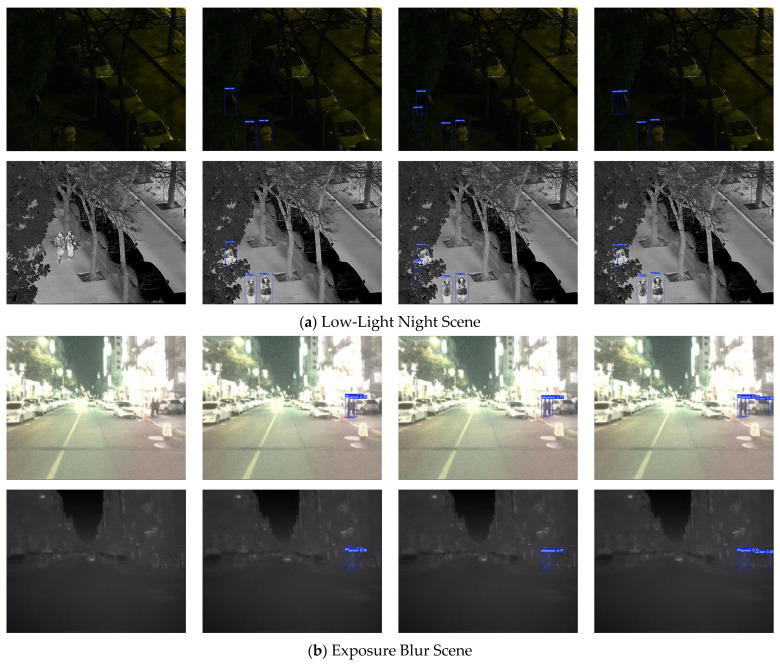
Qualitative comparison under challenging illumination scenes. Each group shows RGB and thermal images with detection results from Dual-YOLOv11s, E2E-MFD, and F^2^Net. Detection boxes are shown with class labels and confidence scores.

Figure 4 presents representative detection results of Dual-YOLOv11s, E2E-MFD, and F^2^Net under low-light night scenes and exposure blur. These cases are used to qualitatively examine whether the proposed fusion strategy can maintain stable target responses when visible details are weakened. Each group includes RGB and thermal images, which allows the response differences among different methods to be compared under challenging illumination conditions. Since this part is a qualitative analysis, it is used as supplementary evidence rather than a substitute for illumination-specific AP evaluation.

In the low-light night scene, the overall illumination of the RGB image is weak, and the grayscale contrast between pedestrians and the background is substantially reduced. As a result, stable target responses are difficult to obtain when relying mainly on visible cues. As shown in Figure 4a, Dual-YOLOv11s suffers from evident missed detections and fails to completely detect distant pedestrians. E2E-MFD detects part of the targets, but several pedestrian regions are still not accurately covered. By contrast, F^2^Net produces more complete detection boxes, with more continuous responses over pedestrian regions. This indicates that, when visible information is strongly degraded, the saliency cues provided by the thermal infrared modality can serve as an effective supplement for target detection.

In the exposure blur scene, strong light sources cause local overexposure in the RGB image, which disturbs object edges and contour information. The target response in the thermal infrared image is also relatively weak. As shown in Figure 4b, both Dual YOLOv11s and E2E-MFD exhibit missed detections or unstable localization in the target regions, whereas F^2^Net maintains clearer target responses and more stable bounding box positions. This improvement is closely related to cross-modal frequency complementary modeling. Overexposure interference in the RGB branch is partially suppressed during fusion, while the relatively stable contour information from the thermal infrared branch provides compensation for target localization.

Overall, Figure 4 shows that F^2^Net is more stable when visible texture is degraded by low illumination or overexposure. The improvement is reflected in more complete target boxes and more stable localization. This result is consistent with the role of frequency-band fusion in suppressing coarse background responses and retaining contour-related cues. It provides qualitative support for the quantitative comparisons, but should not be regarded as illumination-specific quantitative evidence.

**Figure 5:**
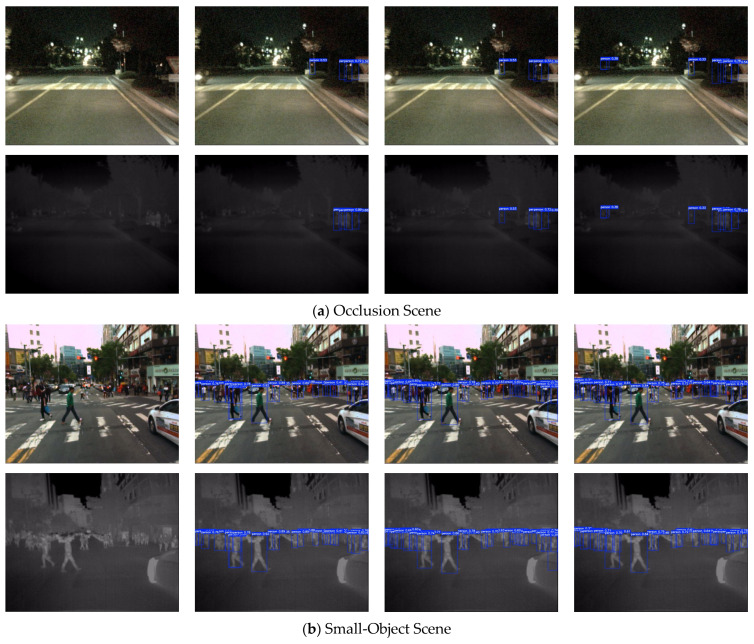
Qualitative comparison under occlusion and dense small-object scenes. Each group includes RGB image, thermal image, ground truth, Dual-YOLOv11s, E2E-MFD, and F^2^Net. Detection boxes are shown with class labels and confidence scores.

As shown in Figure 5, the occlusion scene contains incomplete pedestrian regions and strong foreground interference. Dual-YOLOv11s and E2E-MFD show missed detections or shifted boxes, while F^2^Net responds more stably around target heat-source regions. This improvement is related to the low-frequency reliability mixing in DBCM, which assigns larger weights to the modality with more stable structural cues. In the dense small-object scene, adjacent pedestrians are close to each other and local responses overlap. F^2^Net detects more local targets and keeps clearer boundaries than the compared methods. This observation is consistent with the high-frequency cross-modal gating design, which strengthens boundary-related and small-scale detail responses. These results support the quantitative comparisons, but they remain qualitative evidence rather than a substitute for scale-specific AP evaluation.

Failure analysis under difficult correspondence. Although F^2^Net improves detection stability in low-light, occlusion, and dense scenes, its performance may still degrade when RGB and thermal images exhibit severe spatial displacement or parallax. In such cases, the same spatial location in the two modalities may correspond to different object parts or backgrounds. The saliency-weighted correspondence loss can reduce feature drift under normal registration, but it cannot explicitly correct large geometric offsets. As a result, frequency-guided fusion may enhance inconsistent responses, leading to shifted boxes or missed detections. This limitation indicates that severe RGB-T misalignment requires explicit geometric alignment or deformation-aware fusion in future work.

### 4.8. Heatmap Visualization Analysis

The heatmaps are generated from the final neck feature maps before the detection head by averaging the channel-wise activation responses and normalizing them to the range [0, 1]. The same feature scale and normalization strategy are used for all compared methods. The resulting activation maps are overlaid on the corresponding RGB and thermal images for visualization. Therefore, the heatmaps reflect the relative spatial response of the fused detection features rather than independent RGB-branch or thermal-branch attention.

**Figure 6:**
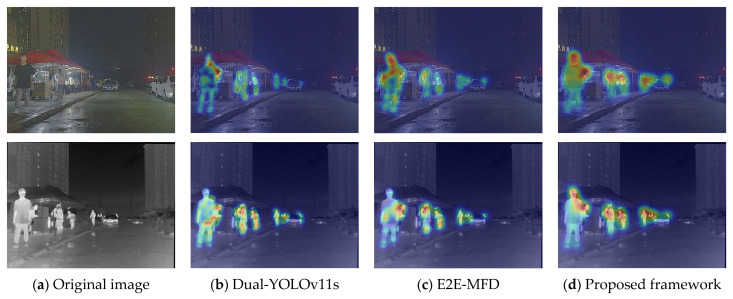
Feature response heatmaps in a typical nighttime scene. The heatmaps are generated from the final neck features before the detection head and normalized to the range from 0 to 1 for all compared methods.

Figure 6 compares the feature response heatmaps of three dual-modal algorithms in a typical night scene. In the original images, the RGB view suffers from insufficient illumination, while the thermal infrared image provides clearer contrast between pedestrians and the background. Dual-YOLOv11s and E2E-MFD show relatively scattered responses, with some activations appearing on background regions or light sources. In contrast, F^2^Net produces more concentrated responses around pedestrian regions and reduces redundant background activations. These observations suggest that the proposed fusion strategy helps the model focus more consistently on target-relevant regions under low-light conditions. Compared with the scattered background activations of the baseline methods, the more concentrated responses of F^2^Net provide visual evidence that frequency-guided fusion suppresses redundant background components and strengthens pedestrian-related feature responses.

## 5. Discussion

The experimental results show that F^2^Net is most beneficial when RGB texture cues are unreliable and thermal responses provide complementary target saliency. This is reflected by the improvements on M3FD and the additional gains observed on LLVIP and KAIST under their respective evaluation protocols. However, the performance pattern also reveals an important limitation: the improvement is stronger for mAP@0.5 than for high-IoU localization metrics, especially on KAIST. This suggests that the proposed frequency-guided fusion improves target response generation more clearly than precise boundary regression in dense and occluded road scenes.

F^2^Net still has limitations in high-precision localization and severe cross-modal misalignment. The current frequency-guided design mainly improves target response generation and detail preservation, while fine-grained boundary regression under heavy occlusion remains difficult. In addition, DCFL assumes reasonably registered RGB-T pairs and does not explicitly correct large spatial displacement or parallax. Future work will combine frequency-guided fusion with deformation-aware alignment and boundary-sensitive regression to further improve high-IoU localization.

The FISRU comparison further shows that frequency-informed reconstruction achieves the best precision, recall, mAP@0.5, and mAP@0.5:0.95 among the evaluated neck reconstruction settings. This indicates that FISRU provides more effective compensation than ordinary nearest-neighbor and bilinear upsampling. The gain mainly comes from regularized frequency compensation, which helps preserve detail-related responses weakened during neck reconstruction. Bilinear interpolation is used only as a tractable frequency prior and does not fully describe the spectral behavior of the whole YOLO neck. The actual neck also contains concatenation, convolution, and feature aggregation, whose combined effect is more complex than a single interpolation kernel. Therefore, FISRU should be viewed as a practical compensation unit for attenuated middle- and high-frequency responses rather than an analytical inversion of the neck. Further spectrum-level visualization and comparisons with generic spatial detail-enhancement modules would provide a more fine-grained verification of the reconstruction strategy.

The current evaluation still has limitations in fine-grained quantitative analysis. The LLVIP and KAIST results, together with qualitative visualizations, provide supporting evidence under low-light, occlusion, and dense small-object scenes. More detailed per-class AP, scale-specific AP, illumination-specific evaluation, occlusion-level evaluation, and controlled RGB-T misalignment tests would further clarify how the gains are distributed across categories, object scales, and spatial displacement conditions.

The ablation results further indicate that the modules are not equally effective in isolation. FISRU and the correspondence regularization loss form the strongest two-component combination, implying that reconstructed high-frequency cues require spatially consistent cross-modal representations to benefit detection. DBCM further improves the complete model by reducing frequency-band interference during early fusion. This result supports the necessity of combining fusion-stage modulation, decoding-stage reconstruction, and training-stage correspondence regularization.

The three datasets also correspond to different practical RGB-T detection scenarios. M3FD represents multi-class traffic and surveillance scenes under daytime, nighttime, fog, glare, and complex illumination, and is suitable for evaluating pedestrians, cyclists, and vehicles in mixed road environments. LLVIP mainly reflects low-light pedestrian monitoring, such as night streets, entrances, parking areas, and public security scenes, where visible images often lose texture and thermal images provide stronger target saliency. KAIST is closer to vehicle-mounted or roadside pedestrian detection, with dense targets, occlusion, and background interference. Therefore, the experiments cover multi-class traffic monitoring, low-light pedestrian surveillance, and road-scene pedestrian detection. These settings provide practical evidence for the proposed fusion strategy, although deployment on embedded RGB-T sensor platforms still requires further testing.

Although F^2^Net improves detection performance under the evaluated protocols, several limitations remain. The current evaluation reports real inference latency, FPS, and peak memory consumption on RTX4060Ti, but edge-oriented deployment has not yet been fully examined. Since frequency-domain operations may have different acceleration and memory-access behavior on edge devices, deployment efficiency should be further evaluated on practical embedded platforms. The current experiments also assume reasonably aligned RGB-T image pairs. Under severe cross-modal misregistration, the same spatial location in RGB and thermal images may correspond to different object regions, and DCFL cannot explicitly correct this geometric offset. Therefore, the current method should be regarded as a frequency-guided fusion framework for normally registered RGB-T pairs rather than a complete solution for severe parallax or spatial distortion. In addition, the evaluations on LLVIP and KAIST are used to examine cross-scene robustness, but they do not constitute strict zero-shot cross-dataset generalization. Future work will focus on lightweight frequency-domain operators, misalignment-robust RGB-T fusion, and real-time deployment evaluation.

## 6. Conclusions

This paper presents F^2^Net, a frequency-guided RGB-T object detection framework for reducing frequency-band interference during cross-modal fusion and detail attenuation during neck decoding. Instead of relying on direct spatial-domain feature fusion, F^2^Net separates coarse structural responses from high-frequency detail cues, reconstructs attenuated spectral components in the neck, and regularizes RGB-T spatial correspondence during training. Experiments on M3FD show clear improvements over the Dual-YOLOv11s baseline with a moderate increase in parameters and GFLOPs. Additional evaluations on LLVIP and KAIST show competitive performance under dataset-specific low-light and road-scene protocols. The results indicate that F^2^Net mainly improves target response generation and moderate-IoU detection. High-precision localization, severe cross-modal misalignment, fine-grained quantitative evaluation, and edge-device deployment remain open issues for future work.

## Figures and Tables

**Table 1:** Effect of the trade-off parameter β on the fusion loss and detection performance.

β	L_align	L_comp	L_fusion	mAP@0.5	mAP@0.5:0.95
0	0.282	0	0.282	0.679	0.528
0.1	0.271	1.873	0.458	0.756	0.594
0.2	0.264	0.835	0.431	0.811	0.642
0.3	0.260	0.514	0.414	0.844	0.671
0.35	0.260	0.430	0.410	0.852	0.678
**0.4**	**0.260**	**0.371**	**0.409**	**0.855**	**0.680**
0.45	0.262	0.330	0.41	0.852	0.678
0.5	0.264	0.301	0.414	0.844	0.671
0.6	0.271	0.266	0.431	0.811	0.642
0.8	0.296	0.251	0.497	0.679	0.528
1	0.336	0.271	0.607	0.459	0.338

**Table 2:** Ablation results of the proposed modules on the M3FD dataset. Bold values indicate the best results.

Model	DBCM	FISRU	DCFL	P/%	R/%	mAP@0.5/%	mAP@0.5: 0.95/%	Param/M	GFLOPs/G
Dual-YOLOv11s				84.7	72.3	81.9	55.5	13.8	33.9
+DBCM(A)	√			86.8	74.7	83.2	56.0	14.5	34.8
+FISRU(B)		√		87.7	75.5	83.7	56.3	14.2	34.3
+DCFL(C)			√	87.4	74.5	84.1	56.7	13.8	34.0
A + B	√	√		87.8	76.3	85.6	57.1	15.1	35.2
A + C	√		√	88.2	74.8	86.1	57.8	14.5	34.9
B + C		√	√	89.3	77.6	87.0	58.6	14.2	34.5
**F^2^Net**	√	√	√	**91.5**	**80.3**	**89.6**	**62.1**	**15.4**	**35.6**

**Table 3:** Direct comparison between FISRU and standard neck reconstruction settings on M3FD. Bold values indicate the best results.

Reconstruction Setting	P/%	R/%	mAP@0.5/%	mAP@0.5:0.95/%	Param/M	GFLOPs/G
Nearest-neighbor upsampling	86.2	73.5	82.8	55.4	13.8	33.9
Bilinear upsampling	86.8	74.1	83.4	55.9	13.8	33.9
**FISRU**	**87.7**	**75.5**	**83.7**	**56.3**	**14.2**	**34.3**

**Table 4:** Real inference efficiency on RTX4060Ti.

Model	Params/M	GFLOPs/G	Latency/ms	FPS	Peak Memory/MB
Dual-YOLOv11s	13.8	33.9	8.7	115	2486
**F^2^Net**	15.4	35.6	9.3	108	2619

**Table 5:** Detection performance comparison between F^2^Net and reproduced single-modal detectors on the M3FD dataset.

Input	Model Category	Model	mAP@0.5/%	mAP@0.5:0.95/%	Params/M	GFLOPs/G
RGB	Two-stage detector	Faster R-CNN [12]	61.8	40.9	41.5	76.5
	Transformer-based detector	RT-DETR [13]	87.2	58.8	19.9	57.0
	Mainstream YOLO Family	YOLOv5s [30]	78.3	51.9	8.6	24.6
		YOLOv8s [31]	78.9	54.1	10.7	28.8
		YOLOv10s [32]	80.2	54.7	7.8	24.9
		YOLOv11s [33]	81.6	55.2	9.2	21.8
IR	Two-stage detector	Faster R-CNN [12]	60.0	39.7	41.5	76.5
	Transformer-based detector	RT-DETR [13]	83.4	55.6	19.9	57.0
	Mainstream YOLO Family	YOLOv5s [30]	76.5	50.7	8.6	24.6
		YOLOv8s [31]	77.1	52.9	10.7	28.8
		YOLOv10s [32]	78.4	53.5	7.8	24.9
		YOLOv11s [33]	79.8	54.0	9.2	21.8
RGB + IR	**Proposed Method**	**F^2^Net**	**89.6**	**62.1**	**15.4**	**35.6**

**Table 6:** Overall performance comparison of F^2^Net with reproduced dual-modal fusion methods on the M3FD dataset.

Model Category	Model	mAP@0.5/%	mAP@0.5:0.95/%	Params/M	GFLOPs/G
Dual-Stream Baseline	Dual-YOLOv5s	79.2	54.1	13.3	34.6
	Dual-YOLOv8s	79.5	54.6	15.9	41.8
	Dual-YOLOv10s	80.5	55.0	10.8	36.3
	Dual-YOLOv11s	81.9	55.5	13.8	33.9
	Dual-RT-DETR	87.1	58.8	32.4	81.6
Leading Dual-Modal Methods	SuperYOLO [34]	86.8	58.1	28.6	72.4
	MMI-Det [35]	87.8	58.6	28.3	75.1
	E2E-MFD [36]	88.5	59.5	34.5	82.6
	MCOR [37]	87.6	57.9	16.2	38.5
	CFMW [38]	88.1	59.2	22.4	45.2
	WaveMamba [39]	89.1	60.8	26.8	55.4
**Proposed Method**	**F^2^Net**	**89.6**	**62.1**	**15.4**	**35.6**

**Table 7:** Reproduced evaluation results on the LLVIP and KAIST datasets. Bold values indicate the best results.

Model Category	Model	LLVIP	LLVIP	KAIST	KAIST
		mAP@0.5/%	mAP@0.5:0.95/%	mAP@0.5/%	mAP@0.5:0.95/%
Dual-Stream Baseline	Dual-YOLOv5s	86.9	57.3	68.1	27.9
	Dual-YOLOv8s	88.5	57.9	68.8	28.3
	Dual-YOLOv10s	88.6	58.1	67.8	27.5
	Dual-YOLOv11s	88.7	58.2	69.8	28.9
	Dual-RT-DETR	93.1	59.5	75.2	32.9
Leading Dual-Modal Methods	SuperYOLO [34]	93.4	60.8	73.5	31.8
	MMI-Det [35]	94.2	60.8	74.1	31.5
	E2E-MFD [36]	94.9	61.6	73.2	31.1
	MCOR [37]	94.1	60.7	74.4	31.8
	CFMW [38]	93.8	60.2	76.3	33.5
	WaveMamba [39]	95.0	62.1	75.8	33.1
**Proposed Method**	**F^2^Net**	**95.2**	**62.3**	**76.7**	**33.8**

## Data Availability

The data used in this study are publicly available.
